# Putting seedlings on the map: Trade‐offs in demographic rates between ontogenetic size classes in five tropical forests

**DOI:** 10.1002/ecy.4527

**Published:** 2025-01-22

**Authors:** Stephan Kambach, Helge Bruelheide, Liza S. Comita, Richard Condit, S. Joseph Wright, Salomón Aguilar, Chia‐Hao Chang‐Yang, Yu‐Yun Chen, Nancy C. Garwood, Stephen P. Hubbell, Pei‐Jen Luo, Margaret R. Metz, Musalmah Bt. Nasardin, Rolando Pérez, Simon A. Queenborough, I‐Fang Sun, Nathan G. Swenson, Jill Thompson, María Uriarte, Renato Valencia, Tze Leong Yao, Jess K. Zimmerman, Nadja Rüger

**Affiliations:** ^1^ Institute of Biology, Department of Geobotany and Botanical Garden Martin Luther University Halle‐Wittenberg Halle (Saale) Germany; ^2^ German Centre for Integrative Biodiversity Research (iDiv) Halle‐Jena‐Leipzig Leipzig Germany; ^3^ School of the Environment Yale University New Haven Connecticut USA; ^4^ Smithsonian Tropical Research Institute Panama Ancón Panama; ^5^ Field Museum of Natural History Chicago Illinois USA; ^6^ Morton Arboretum Lisle Illinois USA; ^7^ Department of Biological Sciences National Sun Yat‐sen University Kaohsiung Taiwan; ^8^ Department of Natural Resources and Environmental Studies National Dong Hwa University Hualien Taiwan; ^9^ School of Biological Sciences Southern Illinois University Carbondale Carbondale Illinois USA; ^10^ Department of Ecology and Evolutionary Biology University of California, Los Angeles Los Angeles California USA; ^11^ Department of Biology Lewis & Clark College Portland Oregon USA; ^12^ Forest Research Institute Malaysia (FRIM) Kepong Selangor Malaysia; ^13^ Center for Interdisciplinary Research on Ecology and Sustainability (CIRES) National Dong Hwa University Hualien Taiwan; ^14^ Department of Biological Sciences University of Notre Dame Notre Dame Indiana USA; ^15^ UK Centre for Ecology & Hydrology Penicuik UK; ^16^ Department of Ecology, Evolution & Environmental Biology Columbia University New York New York USA; ^17^ Escuela de Ciencias Biológicas, Pontificia Universidad Católica del Ecuador, Aptado Quito Ecuador; ^18^ Department of Environmental Sciences Universidad de Puerto Rico San Juan Puerto Rico; ^19^ Department of Economics University of Leipzig Leipzig Germany

**Keywords:** Barro Colorado Island, ForestGeo forest dynamics plot, Fushan, life‐history strategy, Luquillo, Pasoh, seed size, species coexistence, Yasuní

## Abstract

All species must partition resources among the processes that underly growth, survival, and reproduction. The resulting demographic trade‐offs constrain the range of viable life‐history strategies and are hypothesized to promote local coexistence. Tropical forests pose ideal systems to study demographic trade‐offs as they have a high diversity of coexisting tree species whose life‐history strategies tend to align along two orthogonal axes of variation: a growth–survival trade‐off that separates species with fast growth from species with high survival and a stature–recruitment trade‐off that separates species that achieve large stature from species with high recruitment. As these trade‐offs have typically been explored for trees ≥1 cm dbh, it is unclear how species' growth and survival during earliest seedling stages are related to the trade‐offs for trees ≥1 cm dbh. Here, we used principal components and correlation analyses to (1) determine the main demographic trade‐offs among seed‐to‐seedling transition rates and growth and survival rates from the seedling to overstory size classes of 1188 tree species from large‐scale forest dynamics plots in Panama, Puerto Rico, Ecuador, Taiwan, and Malaysia and (2) quantify the predictive power of maximum dbh, wood density, seed mass, and specific leaf area for species' position along these demographic trade‐off gradients. In four out of five forests, the growth–survival trade‐off was the most important demographic trade‐off and encompassed growth and survival of both seedlings and trees ≥1 cm dbh. The second most important trade‐off separated species with relatively fast growth and high survival at the seedling stage from species with relatively fast growth and high survival ≥1 cm dbh. The relationship between seed‐to‐seedling transition rates and these two trade‐off aces differed between sites. All four traits were significant predictors for species' position along the two trade‐off gradients, albeit with varying importance. We concluded that, after accounting for the species' position along the growth–survival trade‐off, tree species tend to trade off growth and survival at the seedling with later life stages. This ontogenetic trade‐off offers a mechanistic explanation for the stature–recruitment trade‐off that constitutes an additional ontogenetic dimension of life‐history variation in species‐rich ecosystems.

## INTRODUCTION

Following the general principles of life‐history theory, all organisms must partition limited resources among demographic processes related to growth, survival, and reproduction, which leads to trade‐offs between demographic rates that facilitate the local coexistence of species with different life‐history strategies (Lande, 1982; Stearns, 1992). On the global scale, the diversity of plant life‐history strategies tend to be aligned along two main gradients: the fast–slow continuum (Reich, 2014) and a gradient in plant reproductive strategies (Salguero‐Gómez et al., 2016). On the local scale, the study of simultaneous trade‐offs among coexisting and competing life‐history strategies requires large diversity of co‐occurring species, such as can be found in old‐growth grasslands (Lind et al., 2013; Nerlekar & Veldman, 2020) and tropical forest ecosystems which can harbor up to hundreds of coexisting tree species and life‐history strategies (Condit et al., 2006; Ohse et al., 2023; Worthy & Swenson, 2019).

Previous studies have shown that the local diversity of life‐history strategies in tropical forests is often structured along two orthogonal demographic trade‐offs: the well‐known growth–survival trade‐off which separates species with fast growth rates from species with high survival rates (the fast–slow continuum) and the less well‐studied stature–recruitment trade‐off which separates species with tall adult stature from species with high recruitment success (Kambach et al., 2022; Kohyama, 1993; Rüger et al., 2018). However, most of these previous analyses have only included growth and survival of trees ≥1 cm diameter at 1.3 m above ground (dbh, Russo et al., 2021; Wright et al., 2010), thus neglecting how the growth and survival rates of seedlings relate to growth and survival at larger tree size classes.

At Barro Colorado Island (BCI) in Panama, seedling growth and survival were related to both demographic trade‐offs (Rüger et al., 2018); that is, most species that grow fast as seedlings also tend to grow fast as adults while species that survive well as seedlings also achieve high survival at larger size classes (Gilbert et al., 2006; Kunstler et al., 2009). Yet, after accounting for this general trade‐off between growth and survival rates at BCI, the remaining variation in life‐history strategies was structured along an orthogonal gradient that separated species with high recruitment rates and above‐average growth and survival rates at the seedling stage from species with tall adult stature and above‐average growth and survival rates at larger size classes (i.e., ≥1 cm dbh, King et al., 2006; Rüger et al., 2018). However, it is not known whether the observed association between the stature–recruitment trade‐off and a trade‐off in growth and survival rates between seedlings and larger size classes observed at BCI holds at other forest sites.

Another limitation in studies of life‐history strategies in forest ecosystems relates to the quantification of reproduction, which is often represented by recruitment rates over a given stem size threshold (e.g., ≥1 cm dbh). To derive a comparable measure of allocation to reproduction among species, the number of recruits needs to be scaled by either the number of seeds produced, the resources invested into seedling survival, or the number of reproductive trees (Bogdziewicz et al., 2023). Since information on the reproductive status of trees or production of seeds is not often available and allocation to seedling survival cannot be measured at all, recruitment rates can sometimes only be scaled by the number or basal area of conspecific “presumably reproductive” trees (e.g., Kambach et al., 2022; Rüger et al., 2018). This scaling approach hinges on the uncertainty about the size at which species become reproductive and bears the potential to neglect differences between monoecious and dioecious species. To avoid this problem, we collated data on seed production from seed traps and data on seedling establishment from seedling censuses to calculate seed‐to‐seedling transition rates, a measure of reproductive success that does not require any scaling. This allowed us to quantify how seed‐to‐seedling transition rates were related to different axes of life‐history differentiation (Muscarella et al., 2013) and whether seed‐to‐seedling transition rates showed a negative relationship with species maximum stature as predicted by Kohyama (1993).

In trees (as in plant species in general), species' life‐history strategies and thus their position along the demographic trade‐off gradients, are manifested through differences in key functional traits such as plant stature, stem or wood density, seed mass, and leaf specific area (Adler et al., 2014; Díaz et al., 2016). At BCI (Rüger et al., 2018), species' positions along the growth–survival trade‐off gradient were predicted by differences in wood density and seed mass (with lower wood density and lower seed mass being indicative of faster life‐history strategies), while species' positions along the stature–recruitment trade‐off gradient were predicted by differences in maximum height and seed mass (with larger maximum height and seed mass being indicative of lower recruitment rates). While these trait‐demography relationships also tend to hold at the seedling stage (Metz, Wright, et al., 2023) their relative importance can shift with ontogenetic stage (Visser et al., 2016) and we lack a comprehensive understanding of the predictive value of plant traits for life‐history variation across all size classes. An understanding of these trade‐offs is urgently needed to accurately assign tree species into groups of similar form and life‐history strategy (Rüger et al., 2018), which can then be used to predict forest dynamics (Rüger et al., 2020) and species' vulnerability to climate‐ and land‐use change (Ohse et al., 2023).

In this study, we compiled seed, seedling, and tree census data from five tropical forests in Asia and the Americas to calculate species‐level seed‐to‐seedling transition rates as well as growth and survival rates in two seedling size classes (from seed germination <20 cm and 20–50 cm height) and two tree size classes that reflect differences in light availability (overstory, understory). Using principal components and post hoc correlation analyses, we identified the trade‐offs that structure the local diversity in life‐history strategies and their relationships with four key functional traits (maximum dbh, wood density, seed mass, and specific leaf area). We investigated (1) how seedling growth, survival and seed‐to‐seedling transition rates relate to the demographic rates of larger trees and (2) how the four functional traits relate to the main demographic trade‐offs across the full‐size spectrum of trees. Specifically, we asked whether seedling growth and survival rates align with the growth–survival trade‐off for larger trees and to what degree is the stature–recruitment trade‐off associated with a growth or survival trade‐off between ontogenetic stages (i.e., between seedlings and larger tree size classes). The results provided us with a better understanding of the demographic trade‐offs that structure local plant diversity across the full‐size spectrum and improved our understanding of the mechanisms that facilitate species coexistence in diverse plant communities.

## METHODS

### Study sites

We assembled data on freestanding woody plant species from five tropical forest dynamics plots of 16–50 ha from the ForestGeo network (Anderson‐Teixeira et al., 2015; Davies et al., 2021). The five sites are BCI (Panama), Fushan (Taiwan), Luquillo (Puerto Rico), Pasoh (Malaysia), and Yasuní (Ecuador) (Table 1). The forest at Luquillo experienced major cyclonic storms in 1989 and 1998. The forest at Fushan is exposed to major storms annually during the typhoon season from July to September each year and experienced major cyclonic storms in 2004, 2008, and 2013 (Hogan et al., 2018). At Pasoh, the forest understorey was heavily disturbed by a 100‐fold increases in native wild boar populations, which fed on nearby oil‐palm plantations and used the understorey plants to construct their birthing nests (Luskin et al., 2017). The forests at BCI and Yasuní had no major disturbances during the study period. In each forest, individuals <1 cm dbh (dbh 1.3 m from the ground, hereafter seedlings) were mapped and measured in subplots of the ForestGeo plots, and all individuals ≥1 cm dbh (hereafter trees) were mapped and measured following Condit (1998). All five ForestGeo plots have been re‐censused approximately every five years, with different time periods used for this study, as a result of data availability (BCI: 1982–2015, Yasuní: 1996–2008, Pasoh: 1987–2010, Luquillo: 1992–2016, and Fushan: 2004–2013).

**TABLE 1:** Characteristics of the five long‐term forest dynamics plots.

ForestGeo plot (country)	Latitude; longitude	MAT (°C)	Precipitation (mm)	Plot size (ha)	Census dates	Forest type (disturbance type)	Species no.	Allometric coefficients^†^	
								*a*	*b*
Barro Colorado Island, BCI (Panama)	9°9′15.48″; −79°50′45.96″	27.1	2551	50	1982, 1985, 1990, 1995, 2000, 2005, 2010, 2015	Broadleaf evergreen (seasonal drought)	299	0.03615016	1.281928
Fushan (Taiwan)	24°45′41.04″; 121°33′18″	18.2	4271	25	2004, 2008, 2013	Broadleaf evergreen (cyclonic storms)	101	0.01652303	1.3
Luquillo (Puerto Rico)	18°19′34.32″; −65°48′57.6″	22.8	3548	16	1992, 1996, 2001, 2006, 2011, 2016	Broadleaf evergreen (cyclonic storms)	138	0.02477302^‡^	1.320^‡^
Pasoh (Malaysia)	2°58′55.2″; 102°18′46.8″	27.9	1788	50	1987, 1990, 1995, 2000, 2005, 2010	Mixed evergreen dipterocarp (everwet)	814	0.1539492	1.204044
Yasuní (Ecuador)	0°41′9.244″; −76°23′49.2″	28.3	3081	50	1996, 2003, 2008	Broadleaf evergreen (everwet)	1114	0.03615016	1.281928

Abbreviation: MAT, mean annual temperature.

† To calculate crown area (in square meters) = *a* × dbh (in millimeters)^*b*^.

^‡^ Separate coefficients for Arecaceae with *a* = 0.002200656, *b* = 1.647.

For all five forests, we combined seed trap data with recruitment data from annual seedling censuses to calculate seed‐to‐seedling transition rates, and we assembled data on species' maximum dbh, wood density, and specific leaf area. In addition, we assembled data on species seed mass (these data were not available for Pasoh). In the following sections, we have described the measurements and calculations of all species‐level demographic rates and traits.

### Data on growth and survival of trees ≥1 cm dbh

In every census, each individual with at least one living stem ≥1 cm dbh was identified to species or morphospecies, determined as “alive” and the stem with the largest dbh was measured with an accuracy of 1 mm following the protocol presented in Condit (1998). For each individual tree where the main stem survived a census interval, we calculated growth as the absolute annual increment in dbh $\frac{{\mathrm{dbh}}_{t2}-{\mathrm{dbh}}_{t1}}{t}$ with *t* = time in years between consecutive censuses.

To account for the dependence of growth and survival on tree size and light availability, we assigned each tree to the overstory or the understory layer, based on the perfect plasticity approach as described in Kambach et al. (2022). The crown area of each individual tree was approximated with the allometric equation crown area (in square meters) = *a* × dbh (in millimeters)^*b*^. For each species, the allometric coefficients *a* and *b* were either calculated from the Tallo global tree allometry and crown architecture database (for species with ≥10 observations, Jucker et al., 2022) or assigned from site‐specific allometries (listed in Table 1). For BCI and Yasuní, the site‐specific coefficients were extracted from Bohlman and Pacala (2012). For Fushan, the coefficients were calculated from 1228 individuals from 65 tree species (P.J. Luo & C.‐H. Chang‐Yang, unpublished data). For Luquillo, separate coefficients for dicots and monocots were derived from the equations presented in Zambrano et al. (2019). For Pasoh, coefficients were calculated from the six largest individuals from the 100 most abundant species in the 2005 census (S. J. Wright, unpublished data). Each ForestGeo plot was divided into subplots of 31.5 × 31.5 m (following Bohlman & Pacala, 2012), in which we ranked all individuals from the largest to the smallest diameter. We successively assigned the largest individuals to the overstory canopy layer, until their cumulative crown area exceeded twice the subplot area. The remaining smaller individuals were then assigned to the understory canopy layer. This means that trees assigned to the overstory are expected to be shaded by, at a maximum, one other tree crown, whereas trees assigned to the understory are shaded by more than two other tree crowns. Palms (Arecaceae), hemi‐epiphytic, and unidentified species were retained for assignment to the overstory or understory layer but were omitted from all other analyses (this excluded 15 species at BCI, 3 at Fushan, 3 at Luquillo, 46 at Pasoh and 26 at Yasuní).

### Data on growth and survival of seedlings ≤50 cm height

At all five sites, growth, survival, and recruitment rates of seedling individuals <1 cm dbh were measured according to ForestGeo protocols (described in Davies et al., 2021); that is, each seedling was individually tagged, identified to species or morphospecies, deemed dead, missing, or alive, and (in the latter case) its height was measured with an accuracy of up to 1 mm. As our study focused on growth and survival rates during earliest life stages, we only calculated growth and survival rates for two seedling classes: individuals <20 cm height and individuals of 20–50 cm height. Size classes were assigned based on seedling height at the first of two consecutive censuses and independent from seedling height at the second census.

For BCI, we combined measurements from two different datasets. Seedlings <20 cm height were censused annually from 1994 to 2019 (omitting measurements that were more than 1.5 years apart) in 600 seedling plots of 1 m^2^ (Wright et al., 2005, 2015) and seedlings of ≥20 cm height were censused annually from 2001 to 2004, 2008 to 2009, and 2011 to 2013 in ~20,000 evenly stratified 1‐m^2^ seedling plots (Comita et al., 2007, 2023). At Fushan, seedlings were censused quarterly from 2003 to 2010 in 264 seedling plots, and biannually from 2011 to 2019 in 318 seedling plots of 1 m^2^, each one located at a distance of 2 m from 106 focal seed traps (Chang‐Yang et al., 2013). At Luquillo, seedlings were censused annually from 2007 to 2016 in 360 seedling plots of 1 m^2^, each one located at a distance of 2 m around 120 focal seed traps (Muscarella et al., 2013; Zimmerman, 2018). At Pasoh, seedlings were censused annually from 2001 to 2008 in up to 1010 seedling plots of 1 m^2^ (some extra seedling plots were added during this time range), each one located at a distance of 2 m around 336 focal seed traps (Chen, 2007). At Yasuní, seedlings were censused annually from 2002 to 2019 in 600 seedling plots of 1 m^2^, each one located at a distance of 2 m around 200 focal seed traps (Metz et al., 2010).

For each individual seedling that survived a census interval, we calculated growth as the absolute annual increment in height $\frac{{\text{height}}_{t2}-{\text{height}}_{t1}}{t}$ with *t* = time in years between consecutive censuses.

### Data on seed production

Seed fall was recorded in seed traps according to ForestGeo protocols that are described in Davies et al. (2021). At BCI, seeds were collected weekly from 1993 to 2012 in 200 traps of 0.5 m^2^ (Visser et al., 2016; Wright et al., 2005). At Fushan, seeds were collected monthly from 2002 to 2007 in 87 traps and from 2007 to 2019 in 106 traps of 0.5 m^2^ (Chang‐Yang et al., 2024). At Luquillo, we used seed trap data that were collected biweekly from 1993 to 2006 in traps of 0.16 m^2^ and biweekly from 2007 to 2021 in 121 traps of 0.5 m^2^ (shifted to accommodate ForestGeo protocols). At Pasoh, seed were collected weekly from 2001 to 2020 in traps of 0.5 m^2^. We only included data from 2002–2010, 2012–2014, and 2018 as the other years had >2 weeks of missing seed rain observations. At Yasuní, seeds were collected twice each month from 2000 to 2018 in 200 traps of 0.5 m^2^ (Metz et al., 2010).

### Data on species traits

Species‐level trait data were assembled from primary and published sources as follows. For BCI, wood density, specific leaf area, and maximum height measurements are described in Wright et al. (2010) and Rüger et al. (2018). Mean dry seed mass was estimated by averaging measurements from up to five seeds from up to five fruits from up to five individuals in the ForestGeo plot or adjacent areas (Metz et al., 2023; Wright et al., 2010), supplemented with data from Daws et al. (2005). For Fushan, wood density was obtained from plot‐level measurements and from the literature (Chave et al., 2009; Lasky et al., 2013), mean dry seed mass was measured from the seed rain data or obtained from the literature, and specific leaf area was measured on 6–12 individuals of each species and maximum height was measured according to established protocols (Cornelissen et al., 2003). For Luquillo, wood density, mean dry seed mass, specific leaf area, and maximum height measurements are described in Swenson et al. (2012) and Umaña et al. (2016). At Pasoh, wood density for 388 species was extracted from the global wood density database (Chave et al., 2009; Zanne et al., 2009) and specific leaf area and mean maximum height were calculated for the 100 most abundant species (in 2005), as measured from the six largest individuals (based on dbh). At Yasuní, mean dry seed mass was estimated from a pooled sample of seeds from fruiting individuals or seed traps (Fortunel et al., 2016; Metz et al., 2023). Wood density was measured for 2052 trees in 2010 and covers three measurements for 558 species, two measurements for 34 species and single measurements for 16 species. Specific leaf area measurements are described in Kraft et al. (2008).

Maximum dbh was calculated as the mean dbh of the six largest trees of each species at the respective ForestGeo plot. To test if maximum dbh is an appropriate proxy for stature, we tested the relationship between maximum dbh and maximum height in BCI, Fushan, Luquillo, and Pasoh using the Pearson correlation coefficient.

### Calculation of species‐specific demographic rates

For each of the five forests, we estimated species‐specific mean annual growth and survival rates in four size and light availability classes (seedlings <20 cm height, seedlings 20–50 cm height, trees ≥1 cm dbh in the understory and in the overstory canopy) as well as species‐specific mean seed‐to‐seedling transition rates. We only calculated growth and survival rates for species and size classes when there were ≥10 observations.

To speed up computations and to linearize the relationships between variables for principal components analysis, we transformed the observed annual growth rates to an approximate normal distribution using the Modulus transformation with λ = 0.4 (Equation 1) as described in John and Draper (1980) and Condit et al. (2017).

(1) $${\text{growth}}_{\text{trans}}=\left\{\begin{array}{c} \;{\text{growth}}^{\lambda }\;g\geq 0\\ \;-\left\{{\left(-\text{growth}\right)}^{\lambda }\right\}\;g<0 \end{array}.\right.$$

To calculate species‐specific growth rates within each size class, we randomly selected (without replacement) up to 200 growth observations across all census intervals (to speed up computation time) per species and size class. We used hierarchical Bayesian models to calculate the mean transformed growth rate *g* of species *j* in size class *k* with the likelihood of the observed transformed growth rate growth_*i,j*_ of individual *i* of species *j* in size class *k* as follows (Equation 2):

(2) $$\begin{array}{c} {\text{growth}}_{i,j,k}=\text{Normal}\left(g_{j,k},{\text{sigma}}_{j,k}\right)\\ g_{j,k}=\text{Normal}\left(g_{k},\sigma_{g,k}=1\right)\\ {\text{sigma}}_{j,k}=\text{Lognormal}\left({\text{sigma}}_{k},\sigma_{\text{sigma},k}=1\right) \end{array},$$

with *g*_*k*_ and sigma_*k*_ being the size class‐specific average transformed growth rate (across all species) and respective SD (both with uniform flat priors).

To calculate species‐specific survival rates within each size class, we randomly selected (without replacement) up to 1000 survival observations across all census intervals (to speed up computations) per species and size class. We used hierarchical Bayesian models with flat priors to calculate the mean annual survival rate *s* of species *j* with the likelihood of the observed individual *i* of species *j* in size class *k* remaining alive_*i*,*j*_ between consecutive censuses as follows (Equation 3):

(3) $$\begin{array}{c} p\left({\text{alive}}_{i,j,k}|\;s_{j,k}\right)=\text{Bernoulli}\left({\text{alive}}_{j,k}|\;s_{j,k}^{t_{i}}\right)\\ s_{j,k}=\text{Beta}\left(\alpha_{k},\beta_{k}\right) \end{array},$$

with α_*k*_ and β_*k*_ being the size class‐average parameters of the beta distribution. We transformed survival rates to the logarithm of the expected lifespan via $\log\left(\frac{1}{1-\text{survival probability}}\right)$ (based on constant survival probabilities, c.f. Condit, 2022).

To calculate comparable seed‐to‐seedling transition rates, we first calculated, for each forest and census interval, the annual number of newly emerging seedlings per hectare (scaled from the summed area of seedling plots) and the annual number of seeds captured per hectare (scaled from the summed area of seed traps). Seed‐to‐seedling transition rates were then calculated by dividing the number of newly emerged seedlings per hectare per year by the number of seeds captured per hectare per year and averaged across all census intervals. Mean seed‐to‐seedling transition rates >1 were replaced with a value of one. This applied to two species at Luquillo (*Guarea glabra* Vahl, *Syzygium jambos* (L.) Alston), one species at Pasoh (*Xerospermum noronhianum* Blume), and three species at Yasuní (*Clarisia biflora* Ruiz & Pav., *Compsoneura capitellata* (Poepp. ex A.DC.) Warb., and *Crepidospermum rhoifolium* Triana & Planch). We then log‐transformed seed‐to‐seedling transition rates.

### Statistical analyses

We normalized each demographic rate to unit variance (via subtraction of the mean and division by the SD, separately per size class and ForestGeo plot) and used principal components analyses to analyze their joint relationships (both within and across forest sites).

Most species did not have data for all four size classes (Appendix S1: Figure S1), which is a prerequisite for traditional principal components analysis. To be able to include as many species as possible, we retained all species with growth and survival estimates in at least two size classes and used an iterative algorithm to impute missing growth and survival estimates with values that guaranteed a minimized effect on the first four principal components of the available observations (Josse & Husson, 2016). This procedure provides principal components that are robust to the imputation of missing values (Appendix S1: Figures S2–S6).

To test the significance of the resulting principal components, we compared the observed eigenvalues to a distribution of eigenvalues obtained from 1000 random permutations of the demographic rates across species (i.e., against null datasets with no correlation structure, Camargo, 2022). Factors with a loading value of ≥0.3 were determined to be significantly related to the respective principal component. Principal components with significant loadings of growth versus survival rates in opposite directions were considered to capture the growth–survival trade‐off. Principal components with significant loadings of seedling versus tree growth rates or seedling versus tree survival rates in opposite directions were considered to capture the stature–recruitment trade‐off in demographic rates.

For each ForestGeo plot, we quantified the relationships among species functional traits (maximum dbh, wood density, specific leaf area, and seed mass) and the main trade‐offs between growth, survival, and seed‐to‐seedling transition rates (represented by the first two principal components). As trait information were not available for all species (Appendix S1: Figure S1), we determined the significance of these relationships with separate post hoc correlation tests for each trait and applied a Bonferroni‐adjusted significance threshold of *p* < 0.0125.

For each pair of ForestGeo plots, we tested for the similarity in principal components by means of a Procrustes analysis (Legendre & Legendre, 2012), a statistical shape analysis that determines optimal scale, shift, and rotation parameters to compare the distribution of two sets of points in n‐dimensional space. Here, we used Procrustes analyses to test the multivariate correlation (at *p* < 0.05) between the factor loadings of species' demographic rates along the first two principal components between two forest sites.

To quantify global relationships and life‐history variation among the five investigated forests, we conducted a global principal components analysis in which the contribution of each species was weighted according to the inverse of the local species number (to guarantee an equal contribution of each ForestGeo plot). The same imputation method (for dealing with missing data) and significance tests were applied as for the single site analyses (outlined above). Using species' scores along the first and second principal component, we determined the core position and size of the site‐specific local life‐history spaces with minimum convex polygons that covered 10% and 90% of co‐occurring species, respectively.

All analyses were conducted in *R*, version 4.2.2 (R Core Team, 2021), using the following *R*‐packages: rstan and coda to run Bayesian hierarchical models (Plummer et al., 2006; Stan Development Team, 2018), missMDA and PCAtest for data imputation and principal components analysis (Camargo, 2022; Josse & Husson, 2016), ggplot2 for graphical representations (Wickham, 2009), and vegan for post hoc correlation and centroid calculations (Oksanen et al., 2020).

## RESULTS

**FIGURE 1:**
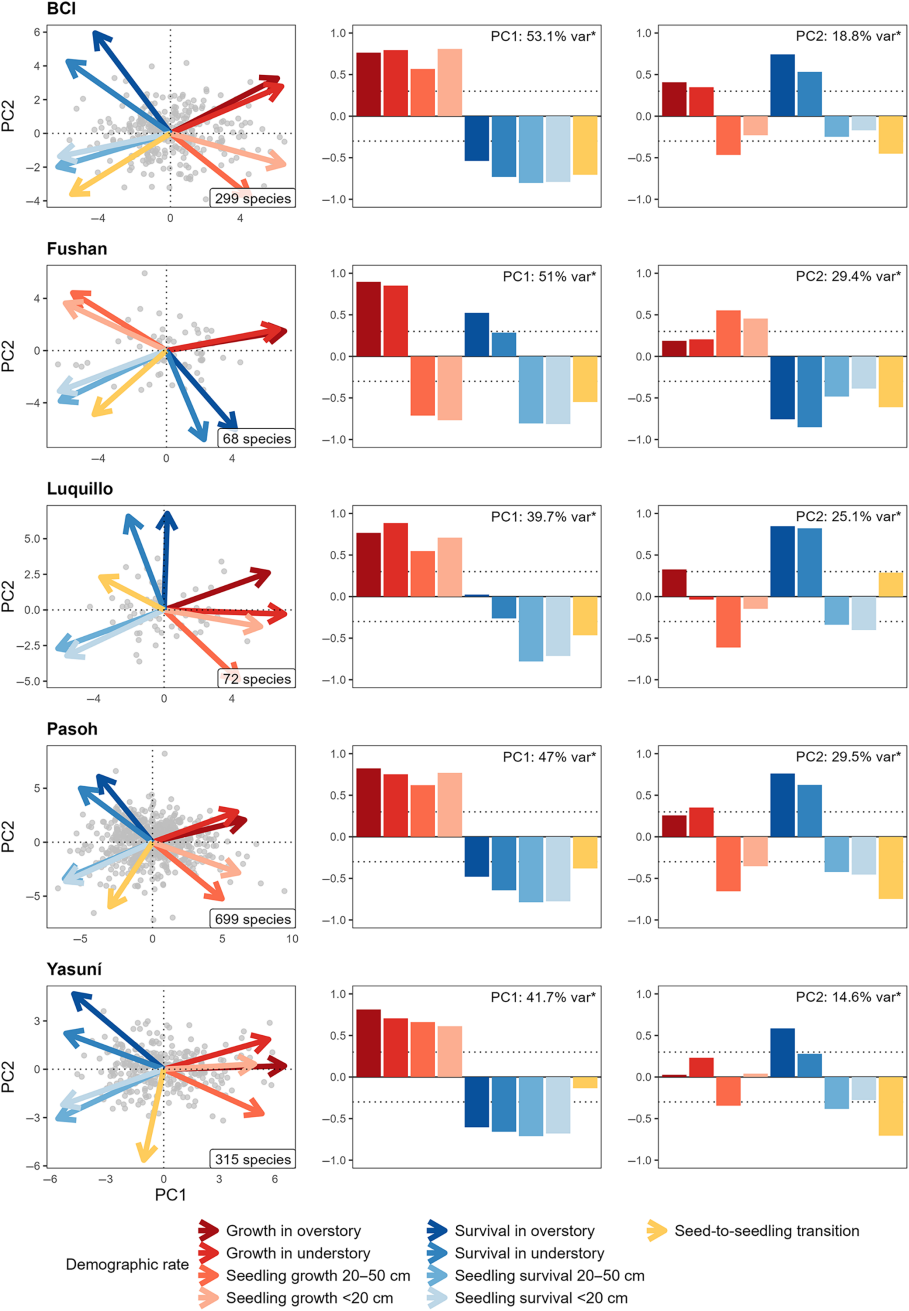
Principal components analyses on species‐specific growth, survival, and seed‐to‐seedling transition rates of tree and shrub species in five tropical forests. Depicted are the joint (arrows) and separate factor loadings (bars) along the first and second principal component. Asterisks indicate significant principal component loadings following permutation tests at *p* < 0.05 (Josse & Husson, 2016).

Growth rates were positively correlated for seedlings and individuals ≥1 cm dbh and survival rates were positively correlated for seedlings and individuals ≥1 cm dbh in three of the five forests (BCI, Pasoh, and Yasuní, Figure 1; pair‐wise correlations are shown in Appendix S1: Figures S7–S11). At Luquillo, seedling growth was likewise positively related to growth of trees ≥1 cm dbh, but survival of seedlings <20‐cm and 20–50‐cm tall was negatively related to survival in the highest canopy layers. At Fushan, however, seedling growth was negatively related to growth of trees ≥1 cm dbh and seedling survival was negatively related to survival in the highest canopy layers.

At each of the five forests, the first two principal components captured a significant proportion of variation in demographic rates and accounted for 56.3%–80.4% of the joint variation in growth, survival, and seed‐to‐seedling transition rates (Figure 1).

Except for Fushan, the first principal component was associated with a general trade‐off between growth and survival rates (growth–survival trade‐off, Figure 1) and the second principal component was associated with a trade‐off between either growth rates, survival rates, or both for the seedling versus tree size classes ≥1 cm dbh (Figure 1). At Fushan, the relative importance of the two trade‐off gradients was switched so that the trade‐off between growth and survival rates at the seedling versus larger tree size classes ≥1 cm dbh was associated with the first principal component and the growth–survival trade‐off was associated with the second principal component.

Across the five forests, seed‐to‐seedling transition rates were consistently positively related to the survival rates of seedlings height <20 cm but showed otherwise varying relationships with species' demographic rates (Appendix S1: Figures S2–S6). Along the principal components of demographic variation, seed‐to‐seedling transition rates were significantly associated with the growth–survival trade‐off at BCI, Fushan, Luquillo, and Pasoh (positively correlated with species' survival rates) and significantly associated with the stature–recruitment trade‐off at BCI, Fushan, Pasoh, and Yasuní (positively correlated with seedling growth and survival rates).

Regarding the similarity in principal components among the five forests, pair‐wise Procrustes analyses showed that the positions of species' demographic rates along the first two principal components were similar among all five forests (with *r* ranging from 0.77 to 0.98 and *p* ranging from 0.008 to <0.001).

**FIGURE 2:**
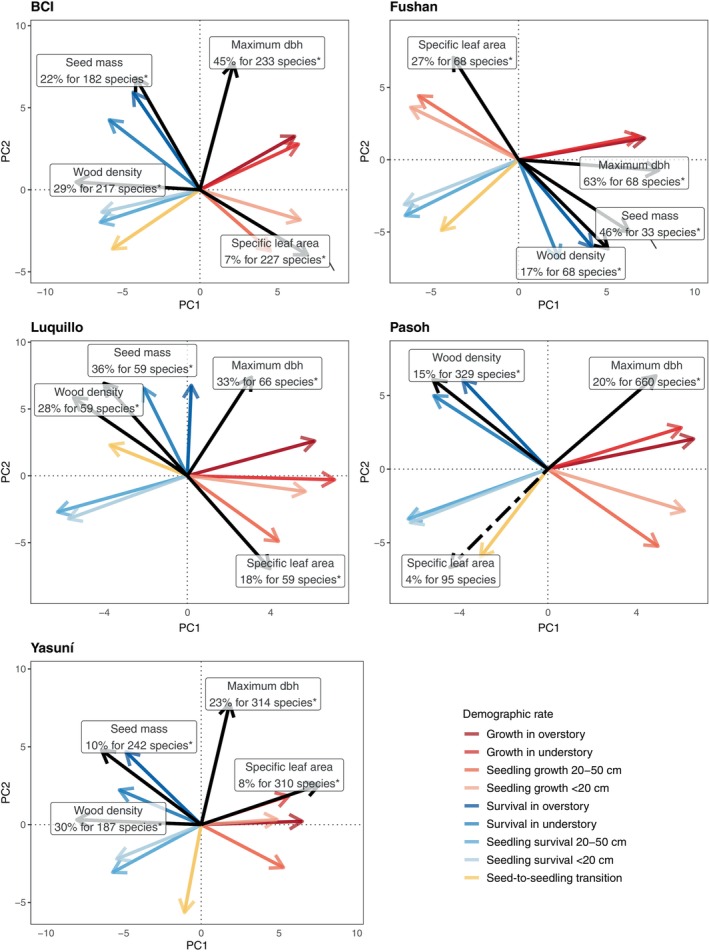
Post hoc correlation tests of four functional traits (black) with the factor loadings obtained from separate principal components analyses of species‐specific growth, survival, and seed‐to‐seedling transition rates (colored) of woody tree and shrub species in five tropical forests (c.f. Figure 1). Black arrows show linear 2D trend surfaces, that is, indicate the direction of the steepest increase in trait values (scaled to unit length) along the first two principal components. Dotted arrows indicate non‐significant relationships between principal components and traits. Text labels show the proportion of explained variation, the number of species, and the Bonferroni‐corrected significance of the post hoc correlation at *p* < 0.015.

The four traits (maximum dbh, wood density, seed mass, and specific leaf area) emerged as significant predictors of species positions along the first two principal components of demographic variation (Figure 2) in all five forests, except for Pasoh where specific leaf area was unrelated to the first two principal components. The relative predictive power of the four traits (inferred from the % of explained variation) differed among the five forests. Maximum dbh (a proxy for maximum height, Appendix S1: Figure S12) accounted for 14%–63% of demographic variation and was positively related to the growth and survival rates of trees ≥1 cm and negatively related to the survival rates of seedlings (Appendix S1: Figures S7–S11). Accordingly, maximum dbh was associated with the trade‐off between growth and/or survival at the seedling versus larger size classes of trees ≥1 cm dbh, indicating a trade‐off between maximum dbh and seedling demographic rates (i.e., the stature–recruitment trade‐off). Wood density accounted for 11%–30% of demographic variation and was most strongly, and positively, related to survival rates ≥1 cm dbh in all five forests. Specific leaf area accounted for 3%–27% of demographic variation and was positively related to seedling growth but negatively related to maximum dbh across all five forests. Seed mass accounted for 10%–46% of demographic variation and positively related to seed‐to‐seedling transition rates and survival of seedlings <20‐cm tall in all four forests with available data (Appendix S1: Figures S7–S9 and S11).

**FIGURE 3:**
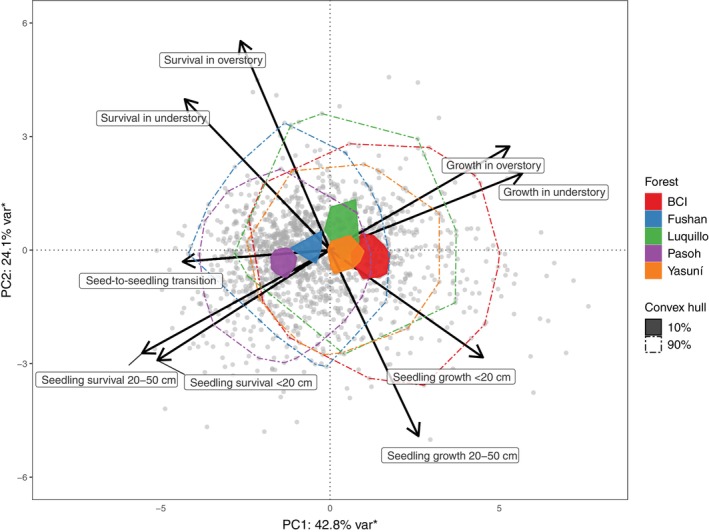
Global principal components analysis and life‐history spaces across the growth, survival, and seed‐to‐seedling transition rates of 1188 tree and shrub species in five tropical forests. Depicted are the joint factor loadings (arrows) and species positions (dots) along the first and second principal component together with the minimum convex hulls that cover the 90% area (dashed lines) and 10% core area (shaded areas) of site‐specific life‐history spaces. Asterisks on axes labels indicate significant principal component loadings following permutation tests at *p* < 0.05 (Josse & Husson, 2016).

When all species‐ and site‐specific demographic rates were analyzed in a global principal components analysis (with equal weights among forests sites), the resulting first and second principal components accounted for 70.7% of the global demographic variation and were associated with the growth–survival and stature–recruitment trade‐offs, respectively (Figure 3). Seed‐to‐seedling transition rates were closely aligned with higher survival along the growth–survival trade‐off. The site‐specific 10% core life‐history spaces mostly aligned along the growth–survival trade‐off, and the 10% core spaces of Pasoh and Fushan were completely separated from the core spaces of BCI, Luquillo and Yasuní. Life‐history spaces that included 90% of all species‐specific life‐history characteristics, however, largely overlapped among the five forests.

## DISCUSSION

In this study, we investigated the main demographic trade‐offs that structured the spectra of life‐history strategies from seedlings to the largest size classes of 1188 tree species in five tropical forests. In four out of the five forests, the growth–survival trade‐off for seedlings and trees was the most important demographic trade‐off (accounting for about 50% of demographic variation, Kambach et al., 2022; Rüger et al., 2018; Russo et al., 2021). After accounting for the growth–survival trade‐off, that is, when comparing only species that had a similar position along the fast–slow continuum, we observed that these species trade‐off investment between growth and/or survival at early life stages (seedlings) versus investment at later life stages (trees). The emergent ontogenetic trade‐off between different life stages provides a mechanistic explanation for the previously reported stature–recruitment trade‐off (Kambach et al., 2022; Rüger et al., 2018), that is, the second orthogonal dimension of life‐history differentiation that separates species with relatively fast growth and high survival at the seedling stage from species that are able to achieve a large maximum stature (Kambach et al., 2022). Relationships of seed‐to‐seedling transition rates with the observed demographic trade‐offs, however, were inconclusive.

Beyond our model system of tropical forests, the growth–survival trade‐off (i.e., the fast–slow continuum of life‐history strategies) has previously been evaluated as a general feature that structures the diversity of plants (Roberto Salguero‐Gómez et al., 2016), fish (Beukhof et al., 2019), mammals (Oli, 2004), and arthropods (Bakewell et al., 2020; Blackburn, 1991). In grasslands, a similar trade‐off can be observed between fast growth versus increased defense against consumers and pathogens (He et al., 2022; Lind et al., 2013). Trade‐offs among growth or survival rates between ontogenetic stages or size classes, however, have less often been investigated. Although pleiotropy theory predicts that finite populations should be structured by a selective pressure that enforces a trade‐off between juvenile and adult survival (Giaimo, 2014), such a trade‐off between ontogenetic stages might be difficult to quantify as it could be masked by the generally stronger signal of the slow–fast continuum. In addition, the relative ecological strategies of plants species are hypothesized to shift with ontogeny‐dependent filters (Dayrell et al., 2018; Wijenayake et al., 2023).

In the cyclonic storm‐disturbed forests at Luquillo and Fushan, the growth–survival trade‐off involved only survival at the seedling stage (Luquillo) or understory size class (Fushan). In contrast to previous studies that focussed on trees ≥1 cm dbh or contrasted growth in high‐resource conditions versus survival in low‐resource conditions (Kambach et al., 2022; Russo et al., 2021; Umaña et al., 2023), the inclusion of seedling census data showed that even the two cyclonic storm‐impacted forests were structured by a growth–survival trade‐off (c.f., Metz et al., 2023) and a stature–recruitment trade‐off (described as a fecundity‐stature axis by Umaña et al., 2023). The life‐history strategies in these two cyclone‐disturbed forests exhibited a negative relationship between survival rates in the seedling stage versus survival rates in the highest canopy layers (described as a survival axis by Umaña et al., 2023). These results highlight that cyclonic storms can condition trade‐offs such as between competitive and colonization ability that are usually not observed in less disturbed tropical forest communities (Kambach et al., 2022; Russo et al., 2021; Umaña et al., 2023; Uriarte et al., 2012).

Although seed‐to‐seedling transition rates showed a consistently positive relationship with seedling survival <20 cm height, the relationships with other demographic rates and trade‐off gradients were inconsistent among the five forests studied. In Pasoh, which is the forest most strongly affected by unnaturally high wild boar populations that caused much disturbance to seedlings in the understorey (Ickes & Thomas, 2003; Luskin et al., 2017), seed‐to‐seedling transition rates were largely decoupled from demographic rates (but not species traits). In the other four forests, we found a negative relationship between maximum stature and seed‐to‐seedling transition rates, which is in line with a mechanism proposed by Kohyama (1993) that enables tree species coexistence in forests with pronounced gap dynamics. We found that species with the fastest growth rates had the lowest seed‐to‐seedling transition rates (except at Fushan), which is consistent with higher seedling mortality associated with smaller seed sizes observed at BCI, Luquillo, and Yasuní (Muscarella et al., 2013; Wright, Calderón, et al., 2024).

In accordance with previous studies, the four plant traits (maximum dbh, wood density, seed mass, and specific leaf area) emerged as significant predictors for the life‐history strategies of tree species (Metz et al., 2023; Rüger et al., 2018; Wright et al., 2010) and explained a comparatively large proportion of demographic variability (c.f. Paine et al., 2015). The specific relationships between traits and growth and survival rates depended strongly on the ontogenetic stage (especially for specific leaf area, seed mass, and maximum height and less so for wood density, Gibert et al., 2016; Lasky et al., 2015). Since leaf and wood traits used for this analysis were measured from individuals ≥1 cm dbh, this could have reduced the strength of their relationships with demographic rates at seedling size classes.

Although our results are based on one of the most comprehensive datasets available on hyperdiverse tree communities, the observed trade‐offs are derived for the rather common species and might have missed fast‐growing species that died or outgrew the 50‐cm seedling height threshold between censuses. One potentially important dimension of life‐history differentiation that we did not explicitly consider includes any potential trade‐offs with fecundity (i.e., biomass production of flowers, fruits, and seeds, Muller‐Landau, 2010; Villellas & García, 2013). Quantifying any trade‐off with the different components of reproduction is still difficult because reproduction effort must be standardized by the local abundance, basal area, or crown area of reproductive individuals (c.f. Kambach et al., 2022) which, to our knowledge, has only been done at BCI (Visser et al., 2016). Here, the Masting Inference and Forecasting network offers a great opportunity to study the global trade‐offs between seed size, seed mass, standardized fecundity, and tree senescence (MASTIF, Qiu et al., 2021, 2022). Finally, our sample size of five forests was too low to investigate how the growth–survival or the stature–recruitment trade‐off might be affected by soil nutrients (Russo et al., 2008), precipitation (Browne et al., 2023), elevation (de Kamimura et al., 2023), biogeographic region (Kambach et al., 2022), or disturbance regimes (Russo et al., 2021). To provide more generalizable patterns across global gradients in climate and biogeography, we encourage the dedicated collection of seedling demographic data with standard protocols in disturbed and undisturbed forests (Davies et al., 2021).

In conclusion, our study adds to the growing body of cross‐site analyses on the trade‐offs that structure the demographic diversity forest ecosystems (Condit et al., 2006; Kambach et al., 2022; Needham et al., 2022; Russo et al., 2021). Beside the well‐documented trade‐off between fast growth and high survival rates, we identified an ontogenetic dimension of life‐history variation that may contribute to coexistence between species that prioritize growth and/or survival at the seedling/juvenile stage and species that prioritize growth and survival at larger size classes. Based on observations from five tropical forests, we hypothesize that these two trade‐offs could pose two general features that structure diverse plant communities.

## AUTHOR CONTRIBUTIONS

Stephan Kambach contributed to conceptualization, data curation, formal analysis, investigation, software, visualization, and writing—original draft preparation. Helge Bruelheide contributed to funding acquisition, conceptualization, data analysis, writing—review and editing. Liza S. Comita contributed to funding acquisition, conceptualization, investigation, writing—review and editing. Richard Condit contributed to conceptualization, investigation, writing—review and editing. Salomón Aguilar, Chia‐Hao Chang‐Yang, Pei‐Jen Luo, Rolando Pérez, Simon A. Queenborough, Nathan G. Swenson, and Tze Leong Yao contributed to investigation, writing—review and editing. Yu‐Yun Chen, Nancy C. Garwood, Stephen P. Hubbell, Margaret R. Metz, Musalmah Bt. Nasardin, I‐Fang Sun, María Uriarte, Renato Valencia, S. Joseph Wright, and Jess K. Zimmerman contributed to funding acquisition, investigation, writing—review and editing. Jill Thompson contributed to funding acquisition, investigation, data curation, writing—review and editing. Nadja Rüger contributed to conceptualization, data curation, funding acquisition, methodology, supervision, writing—review and editing.

## CONFLICT OF INTEREST STATEMENT

The authors declare no conflicts of interest.

## Supporting information


Appendix S1:

## Data Availability

Data are available by plot as follows. For the BCI plot: census data of trees dbh ≥ 1 cm (Condit et al., 2019) available in Dryad at https://doi.org/10.15146/5xcp-0d46; census data for seedling >20‐cm tall (Comita et al., 2023) available in a data paper published in Ecology at https://doi.org/10.1002/ecy.4140; species‐level mean growth and survival rates of seedlings <20 tall (Kambach et al., 2024) available in iDiv, the repository of the German Centre for Integrative Biodiversity Research Halle‐Jena‐Leipzig, at https://doi.org/10.25829/idiv.3565-ws9g97; seed trap data (Wright & Calderon, 2015) available in the Smithsonian Tropical Research Institute repository at https://doi.org/10.5479/si.data.201511251137; trait data (Wright et al., 2016) available in Figshare at https://doi.org/10.6084/m9.figshare.3550359.v1. For the Fushan plot: species‐level mean growth and survival rates of trees dbh ≥ 1 cm, species‐level growth and survival rates of seedlings <50‐cm tall, and species‐level mean annual seed numbers (Kambach et al., 2024) are available in iDiv at https://doi.org/10.25829/idiv.3565-ws9g97; trait data from Iida et al. (2014) at https://doi.org/10.1111/1365‐2745.12221. For the Luquillo plot: census data of trees dbh > 1 cm (Zimmerman, 2023) available in the EDI data portal at https://doi.org/10.6073/pasta/7d937e27dfd99308362049d6c4495deb; census data for seedling >20‐cm tall (Zimmerman, 2018) available in the EDI data portal at https://doi.org/10.6073/pasta/45e4817e74b51b9533b1bd4115415569; seed trap data (Zimmerman & International Institute of Tropical Forestry, USDA Forest Service, 2023) available in the EDI data portal at https://doi.org/10.6073/pasta/c0fb7433617bcd03dfd82bc1fad28cfb; trait data (Swenson & Umana, 2015) available in Dryad at https://doi.org/10.5061/dryad.j2r53. For the Pasoh plot: data on growth and survival rates of trees dbh > 1 cm was collected in collaboration with the Forest Research Institute Malaysia (FRIM) and can only be obtained upon request (see original data request form in Appendix S1: Section S1, which was sent to prcsecretariat@frim.gov.my); species‐level growth and survival rates of seedlings <50‐cm tall (Kambach et al., 2024) available in the iDiv data repository at https://doi.org/10.25829/idiv.3565-ws9g97; and trait data (Wright & Yao, 2024) available in Dryad at https://doi.org/10.5061/dryad.q2bvq83v7. For the Yasuní plot: up to 200 growth observations and up to 1000 survival observations per species and size class dbh > 1 cm (Kambach et al., 2024) are available in the iDiv data repository at https://doi.org/10.25829/idiv.3565-ws9g97; growth and survival rates of seedlings <50 cm tall (Metz, Zambrano, et al., 2023) are available on the EDI data portal at https://doi.org/10.6073/pasta/2cb969b626c3e276770a4fdc8bb3e375; seed trap data (Garwood et al., 2023) are available on the EDI data portal at https://doi.org/10.6073/pasta/5e6cb3d7ff741fd9d21965c4a904bc1f; wood density data (Wright, Alvia, et al., 2024) are available in Figshare at https://doi.org/10.25573/data.25639560.v2; seed mass data (Garwood et al., 2024) are available on the EDI data portal at https://doi.org/10.6073/pasta/95e4095ea61fb3cdc3b29b0cc10fa72e; and specific leaf area data (Kraft & Ackerly, 2010) are available in Figshare at https://doi.org/10.6084/m9.figshare.c.3292639.
