# Supplementary material for: Putting seedlings on the map: Trade‐offs in demographic rates between ontogenetic size classes in five tropical forests
Source: Ecology. 2025 Jan 22;106(1):e4527. doi: 10.1002/ecy.4527 (PMC11755001; doi:10.1002/ecy.4527)
Supplement: Supplementary file 1 — Appendix S1: [file ECY-106-e4527-s001.pdf]

Appendix S1

Putting seedlings on the map: Trade-offs in demographic rates between ontogenetic size classes in five tropical forests

Stephan Kambach, Helge Bruelheide, Liza S. Comita, Richard Condit, S. Joseph Wright, Salomón Aguilar, Chia-Hao Chang-Yang, Yu-Yun Chen, Nancy C. Garwood, Stephen P. Hubbell, Pei-Jen Luo, Margaret R. Metz, Musalmah Bt. Nasardin, Rolando Pérez, Simon A. Queenborough, I-Fang Sun, Nathan G. Swenson, Jill Thompson, María Uriarte, Renato Valencia, Tze Leong Yao, Jess K. Zimmerman, Nadja Rüger

Journal: Ecology

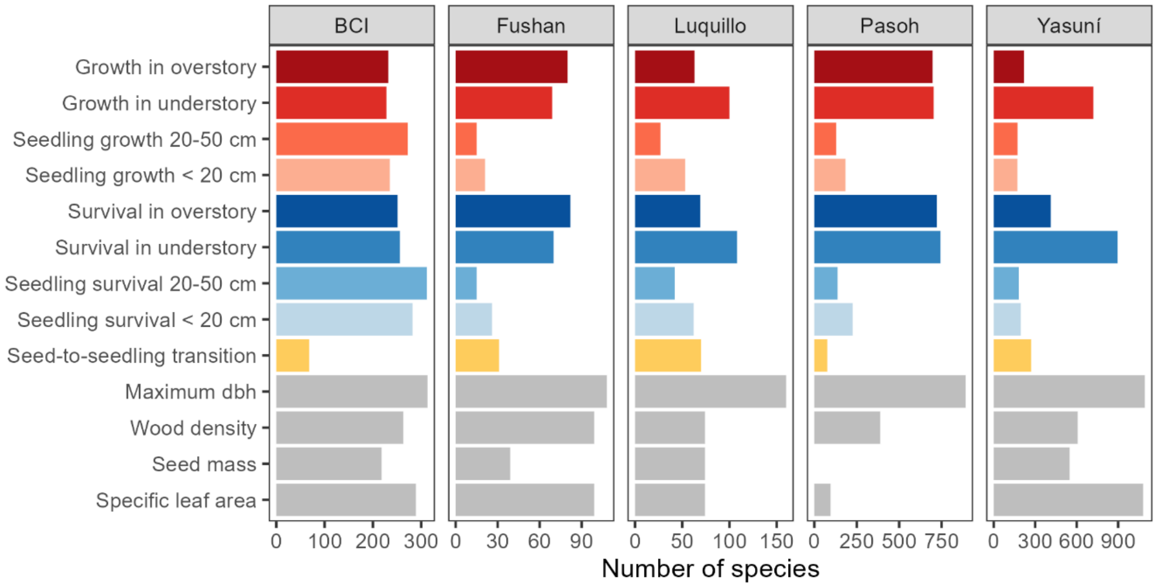

Figure S1. Bar plots showing the number of woody tree and shrub species with estimated demographic rates and traits in five tropical forests. Bars for growth rates (red) and survival rates (blue) show the number of species with at least 10 observations in the respective size class.

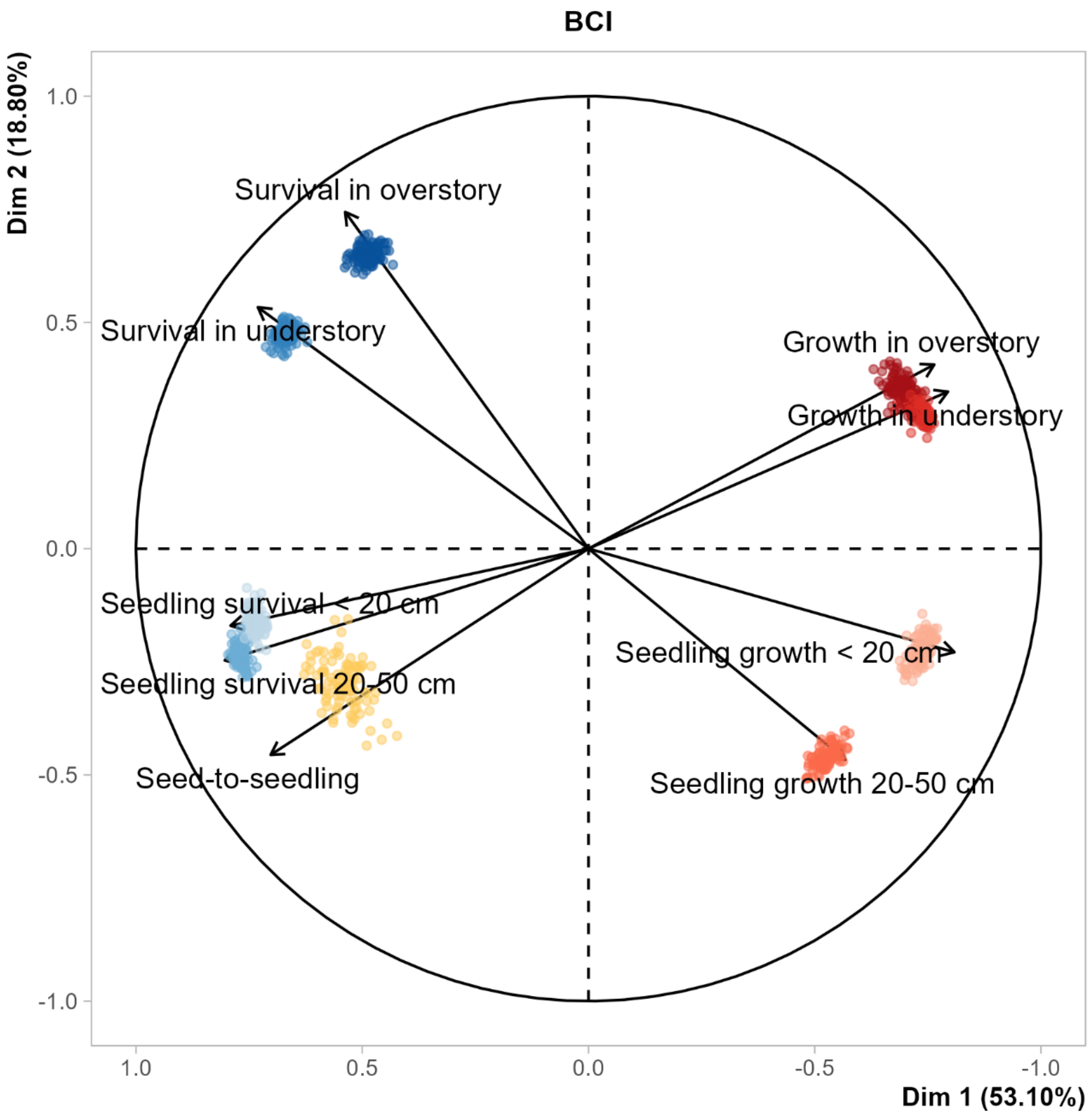

Figure S2. Factor loadings resulting from multiple imputation of missing demographic growth and survival rates at BCI. Arrows show average factor loadings. Points show the individual loadings from 100 imputed datasets along the first and second principal component. A larger spread of individual factor loadings indicates a higher uncertainty around the average factor loadings.

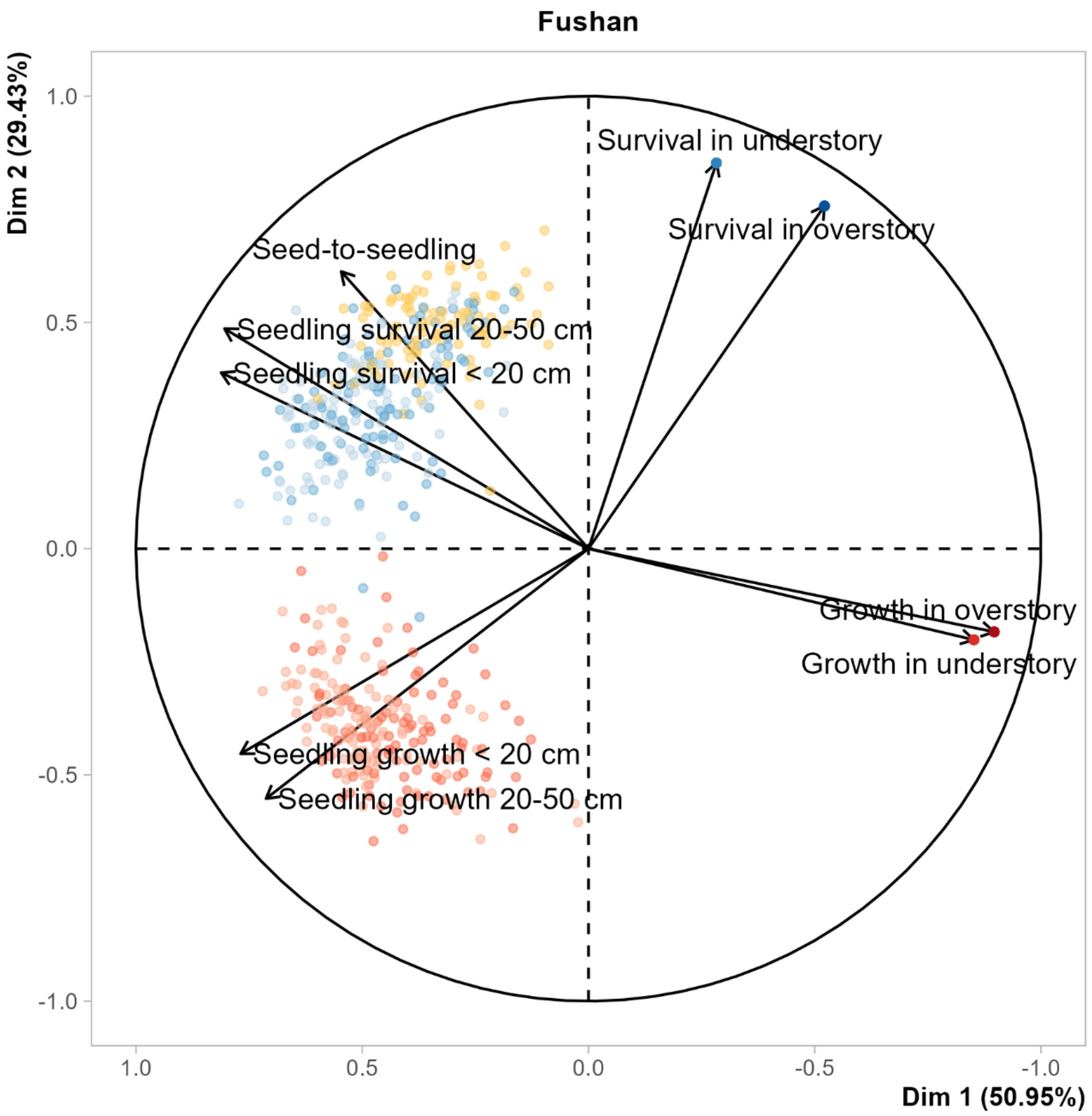

Figure S3. Factor loadings resulting from multiple imputation of missing demographic growth and survival rates at Fushan. Arrows show average factor loadings. Points show the individual loadings from 100 imputed datasets along the first and second principal component. A larger spread of individual factor loadings indicates a higher uncertainty around the average factor loadings.

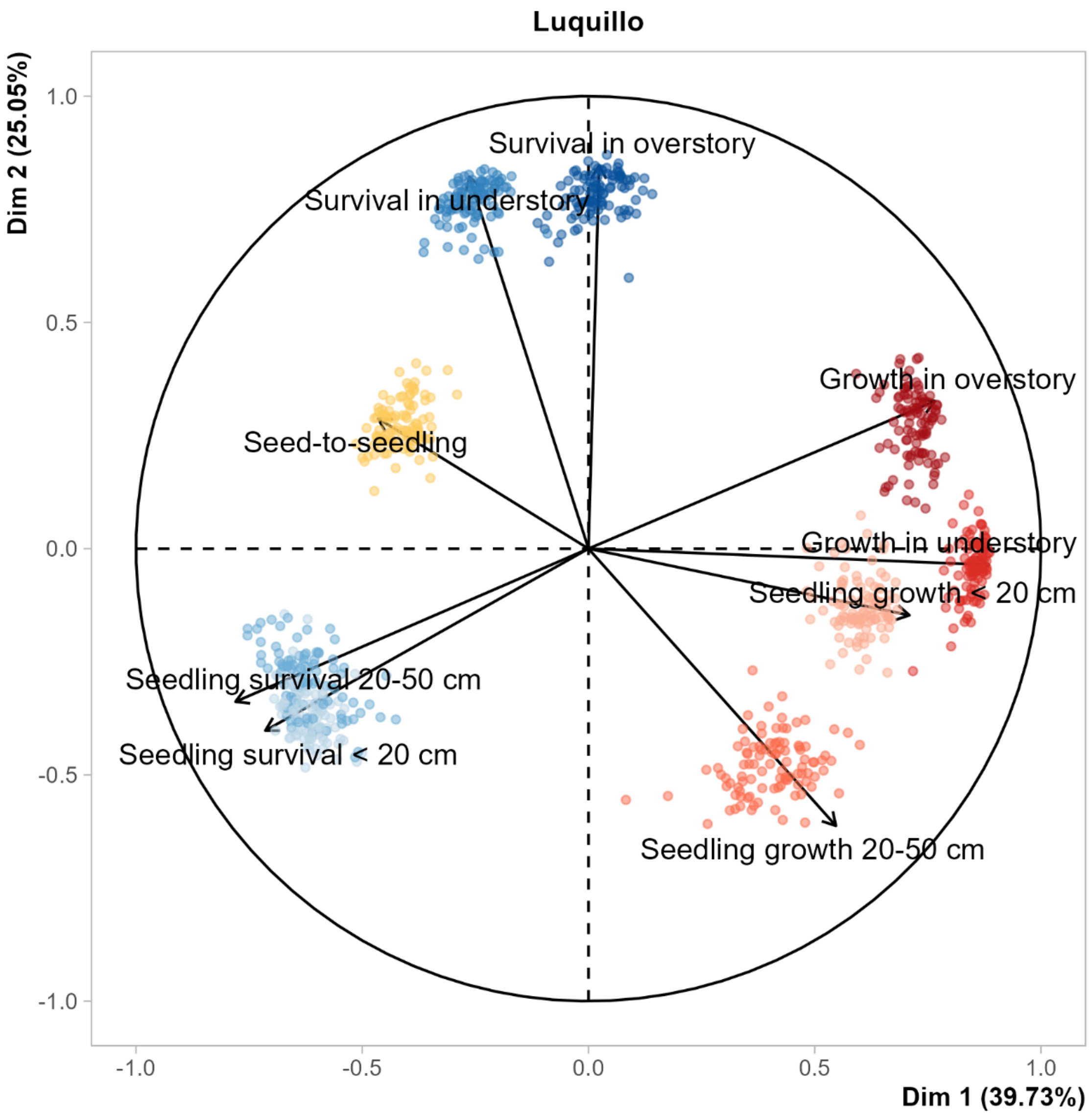

Figure S4. Factor loadings resulting from multiple imputation of missing demographic growth and survival rates at Luquillo. Arrows show average factor loadings. Points show the individual loadings from 100 imputed datasets along the first and second principal component. A larger spread of individual factor loadings indicates a higher uncertainty around the average factor loadings.

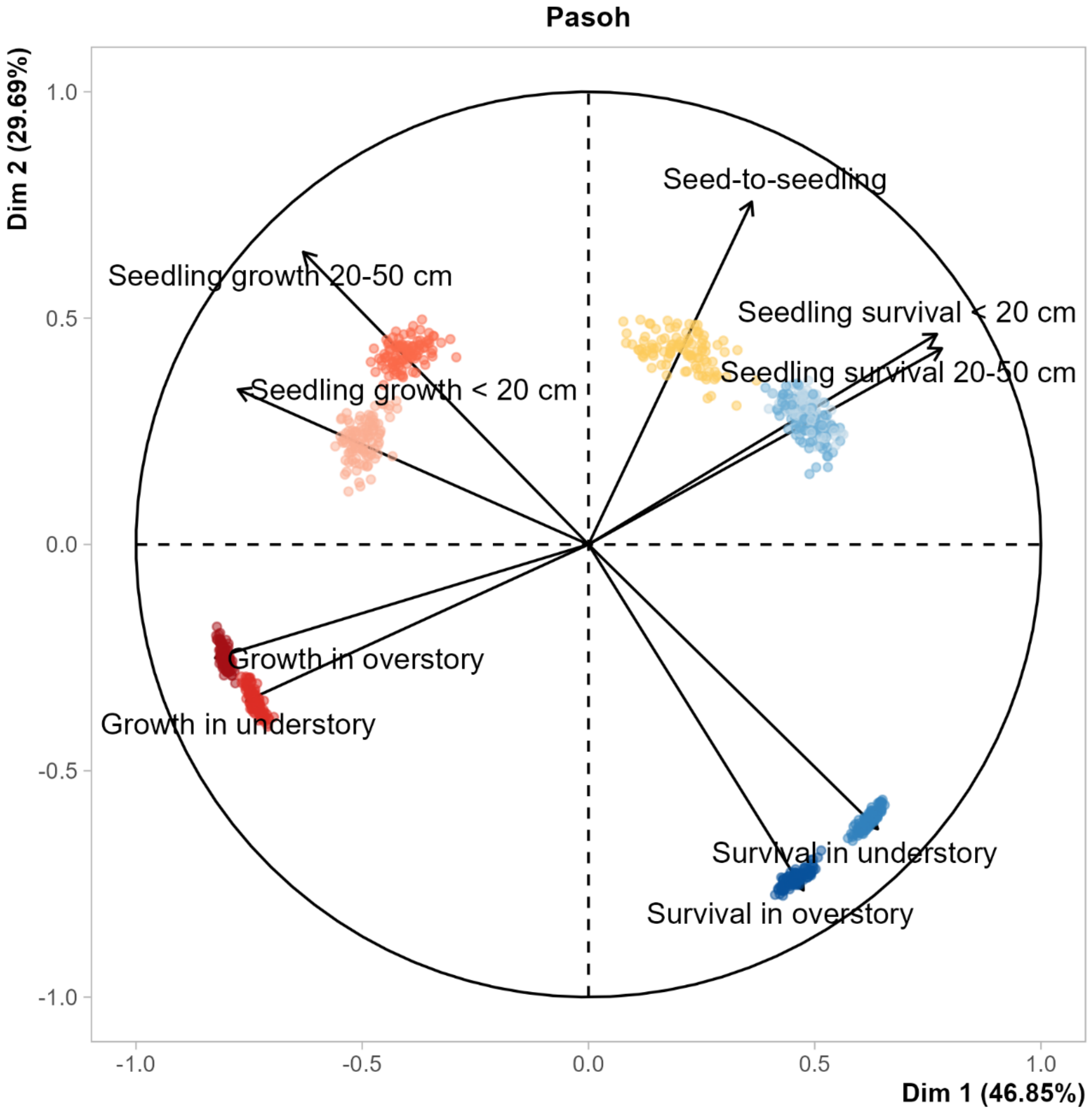

Figure S5. Factor loadings resulting from multiple imputation of missing demographic growth and survival rates at Pasoh. Arrows show average factor loadings. Points show the individual loadings from 100 imputed datasets along the first and second principal component. A larger spread of individual factor loadings indicates a higher uncertainty around the average factor loadings.

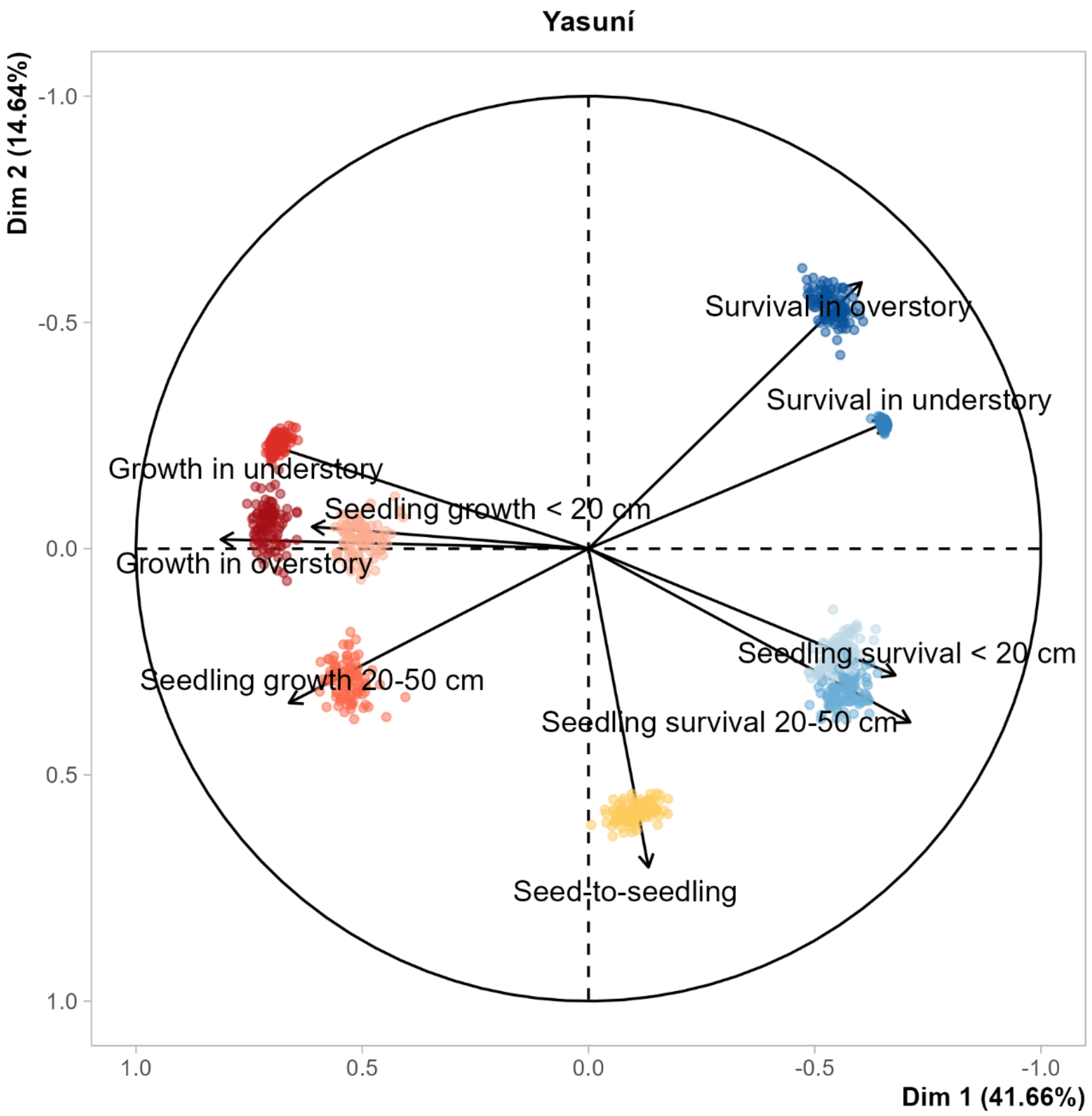

Figure S6. Factor loadings resulting from multiple imputation of missing demographic growth and survival rates at Yasuní. Arrows show average factor loadings. Points show the individual loadings from 100 imputed datasets along the first and second principal component. A larger spread of individual factor loadings indicates a higher uncertainty around the average factor loadings.

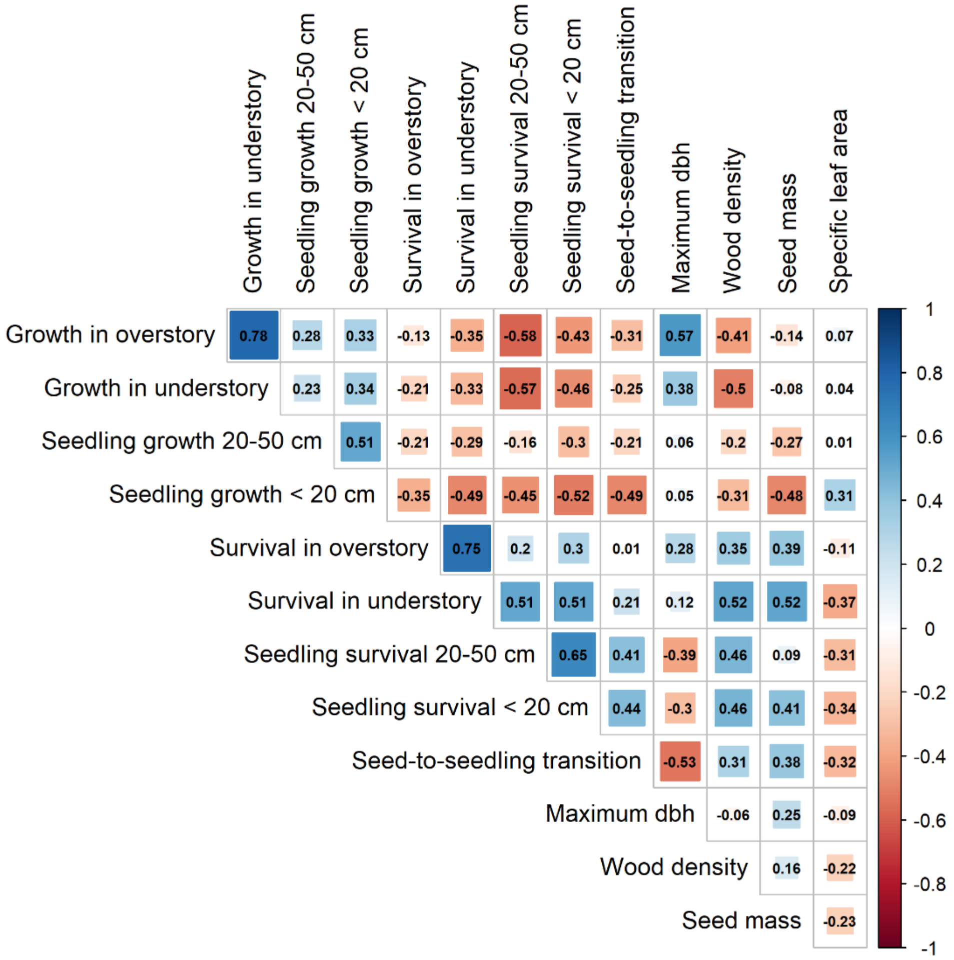

Figure S7. Correlations between species-specific growth and survival rates from four different size classes, together with seed-to-seedling transition rates and species-specific trait values in Barro Colorado Island. Pearson correlations with growth or survival rates include only species with at least ten observations (in the respective size classes).

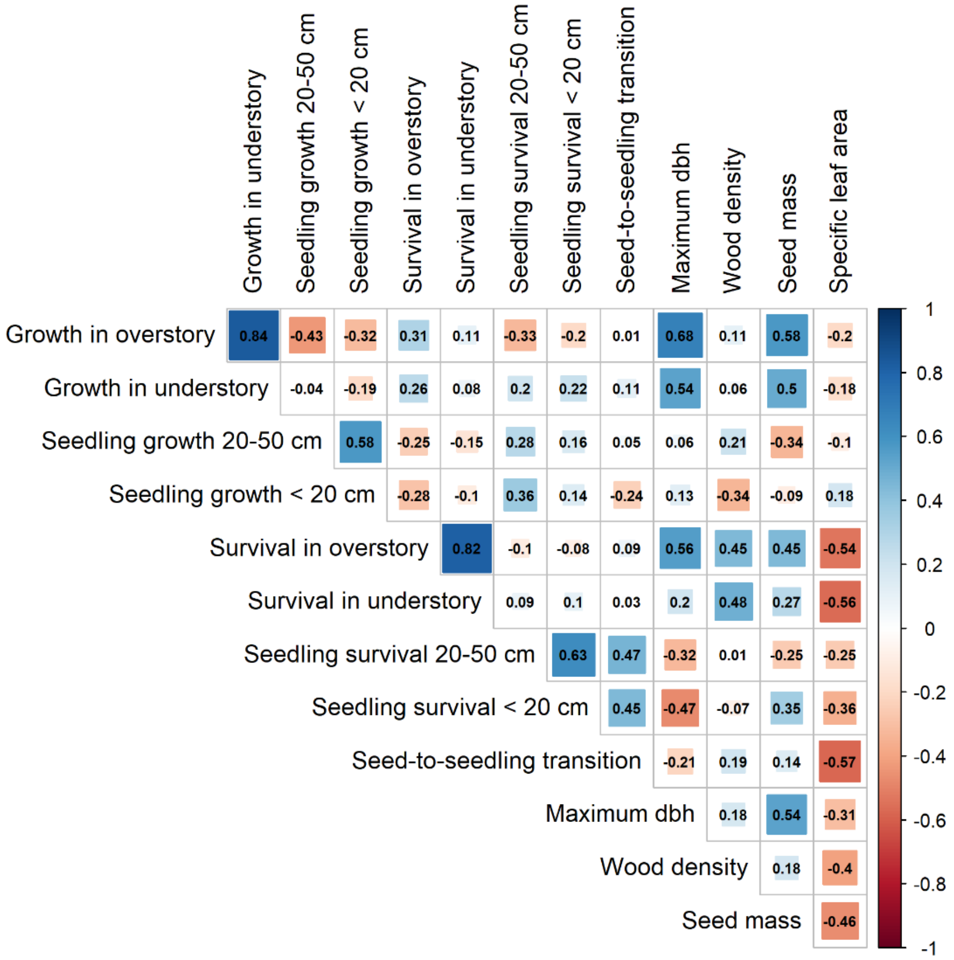

Figure S8. Correlations between species-specific growth and survival rates from four different size classes, together with seed-to-seedling transition rates and species-specific trait values in Fushan. Pearson correlations with growth or survival rates include only species with at least ten observations (in the respective size classes).

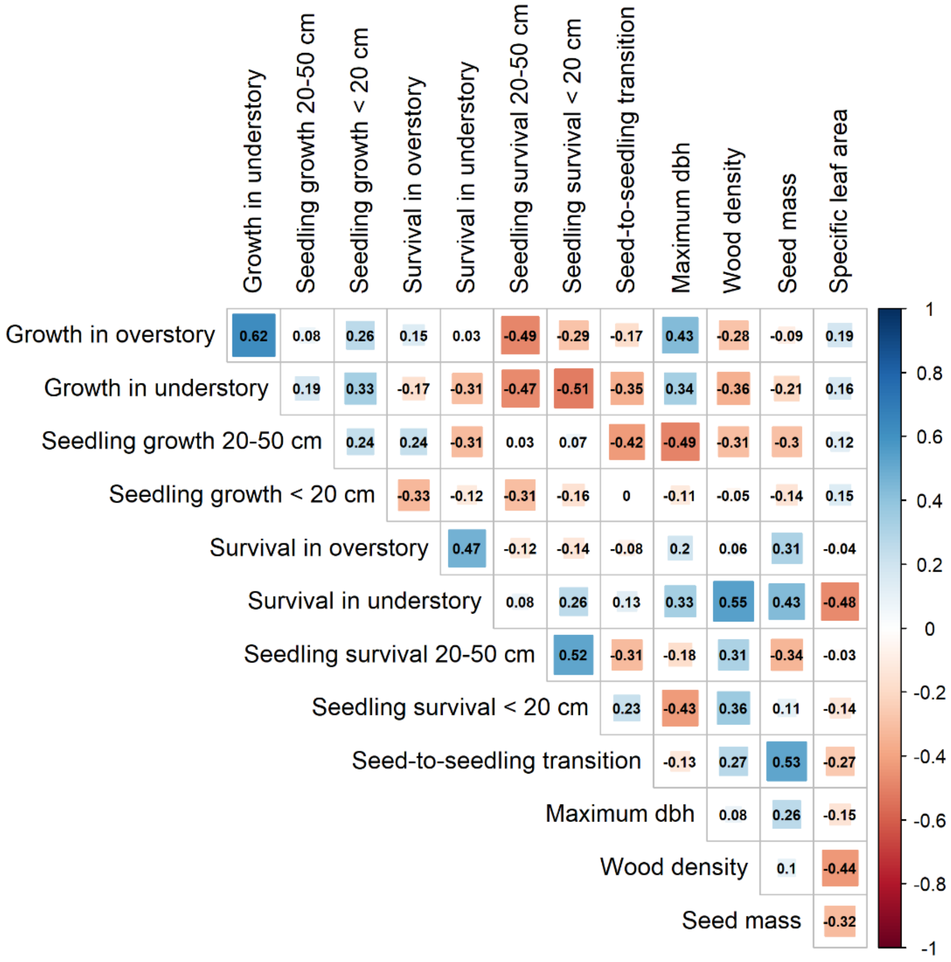

Figure S9. Correlations between species-specific growth and survival rates from four different size classes, together with seed-to-seedling transition rates and species-specific trait values in Luquillo. Pearson correlations with growth or survival rates include only species with at least ten observations (in the respective size classes).

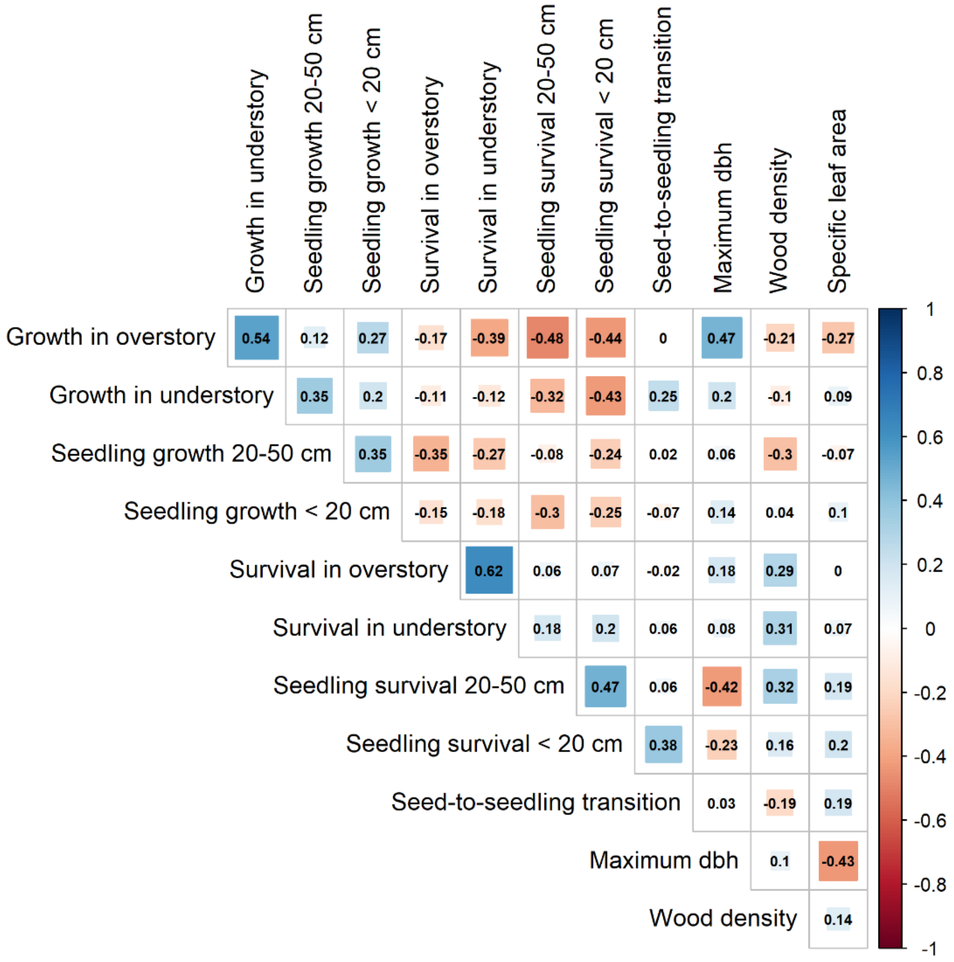

Figure S10. Correlations between species-specific growth and survival rates from four different size classes, together with seed-to-seedling transition rates and species-specific trait values in Pasoh. Pearson correlations with growth or survival rates include only species with at least ten observations (in the respective size classes).

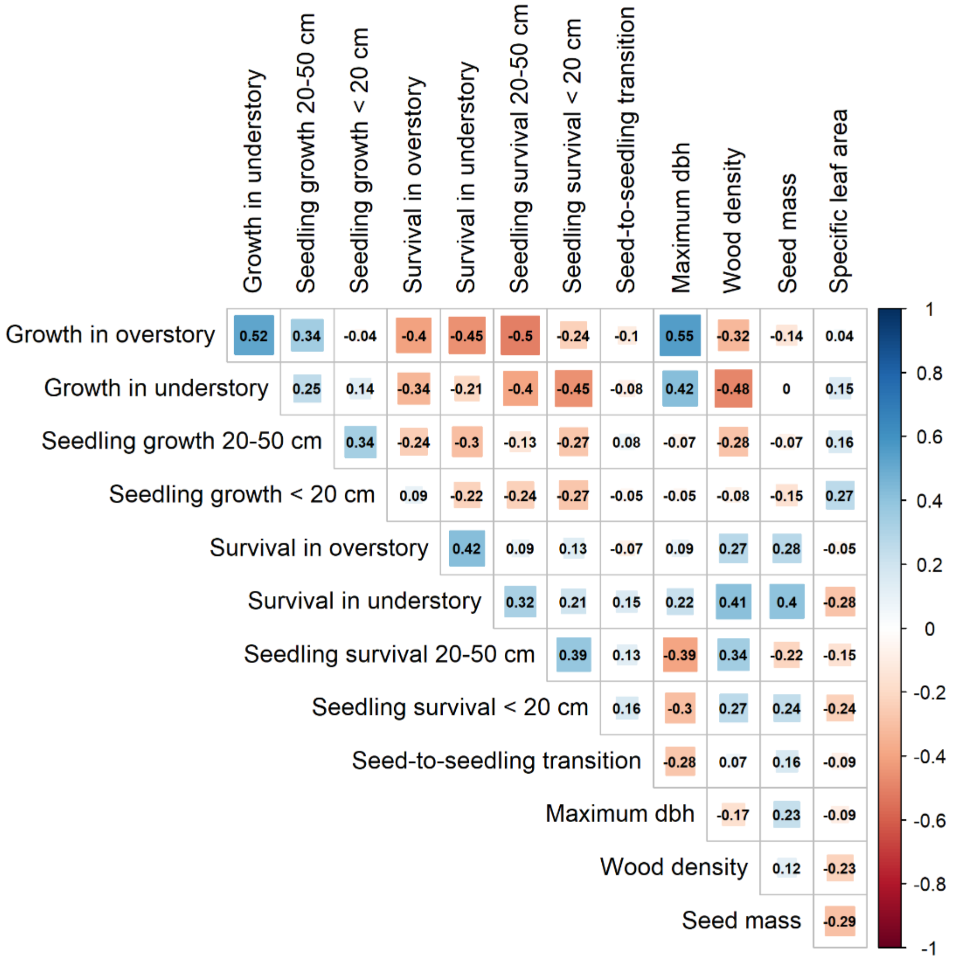

Figure S11. Correlations between species-specific growth and survival rates from four different size classes, together with seed-to-seedling transition rates and species-specific trait values in Yasuní. Pearson correlations with growth or survival rates include only species with at least ten observations (in the respective size classes).

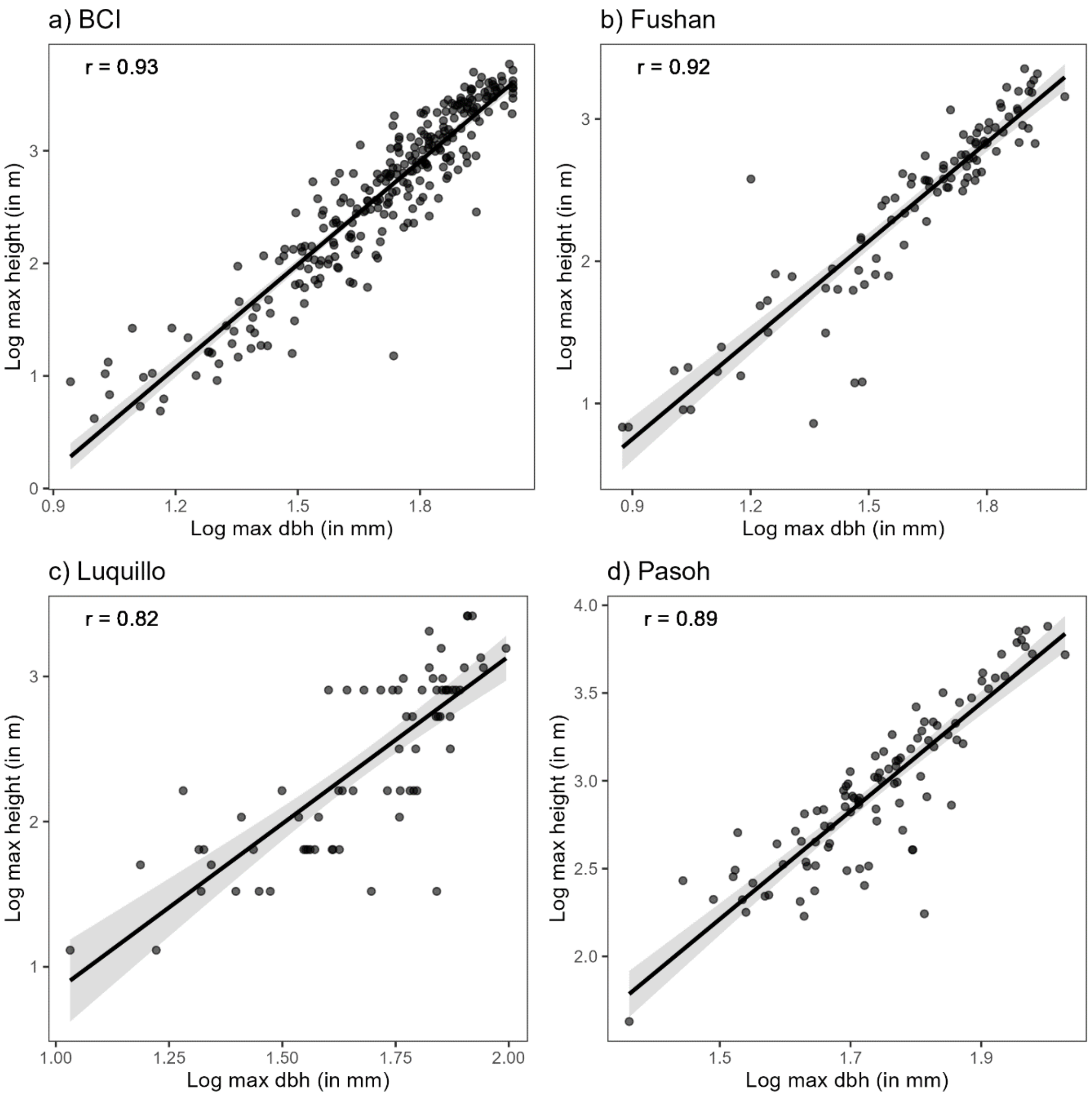

Figure S12. Relationship between log transformed maximum diameter at breast height and maximum height in four tropical forest dynamics plots. Solid lines indicate slopes from linear models and r related to the Pearson correlation coefficient.

Section S1: Data request form submitted to the Pasoh Research Committee (prcsecretariat@frim.gov.my).

PROPOSAL FORMAT FOR PASOH RESEARCH COMMITTEE REVIEW

Project title

Beyond the growth-survival trade-off: A global analysis of demographic diversity and trade-offs in species-rich forests

Project leader and Collaborators

Stephan Kambach[1,2], Nadja Rüger[1,3], S. Joseph Wright[3], Sean McMahon[4] *et al.* (incl. CTFS plot PIs)

1 German Centre for Integrative Biodiversity Research (iDiv) Halle-Jena-Leipzig, Germany

2 Institute of Biology/Geobotany and Botanical Garden, Martin Luther University Halle-Wittenberg, Halle, Germany

3 Smithsonian Tropical Research Institute, Balboa, Ancon, Republic of Panama

4 Smithsonian Environmental Research Center (SERC), Maryland, USA

The project leader, Stephan Kambach, is a graduate student who has already submitted his PhD Thesis. His main supervisor for this proposed project is Nadja Rüger.

Research background

Life history theory posits that organisms face allocation trade-offs underlying different demographic processes (growth, survival, reproduction) and that the resulting trade-offs among demographic rates constrain the range of viable life-history strategies (e.g. Stearns 1999). Recent analyses of demographic trade-offs in >600 vascular plant species across biomes identified two major dimensions of demographic variation: the well-established fast-slow continuum and a novel reproductive strategy dimension (Salguero-Gómez et al. 2016, Salguero-Gómez 2017). However, it remains unclear to what extent these demographic trade-offs also emerge within real-world communities of coexisting species that contain rare species and where all species experience the same climate – a precondition for their relevance to species coexistence (Adler *et al.* 2007).

Objectives

Our overall goal is to develop a holistic understanding of the demographic diversity of trees and shrubs in species-rich forests and how this diversity is shaped by climate, disturbance regime and the floristic composition of the species pool. The specific objectives are (1) to quantify which and how many demographic trade-offs structure diverse forests differing in climate, disturbance regime and floristic composition. We predict that in forests with little disturbance, the fast-slow axis is relatively less and the stature-fecundity axis is relatively more important. (2) We furthermore aim to investigate how demographic trade-offs are constrained by the functional traits of the species in order to elucidate coordinated adaptive strategies at the organ and individual level that allow trees to cope with climatic constraints and the prevailing disturbance regime. This will allow for a simplification of the complex demographic space while retaining key information for subsequent modeling.

Methodology

We will assembled demographic rates from seeds to adult trees and account for tree size and light availability by assigning each individual to one of four canopy layers, based on the number of tree crowns above them (cf. Bohlman & Pacala 2012). We will then estimate growth and survival rates for each tree species in each canopy layer. For subsets of species, we plan to include additional data on seedling growth and survival and, as measures of reproduction, the number of seeds as well as seedling and sapling recruits. We will then determine the major axes of trees demographic diversity using a novel weighted PCA that allows incorporating uncertainty associated with small sample sizes of rare species, which is a crucial feature for application in hyperdiverse systems (Delchambre 2014).

Preliminary findings/expected results

We recently applied the outlined approach to the CTFS plot at Barro Colorado Island (BCI), Panama (Rüger *et al.* in revision) and found that a two-dimensional spectrum of life-history strategies explained already two thirds of demographic variation for the 282 coexisting tree species. In addition to the fast-slow continuum, our results revealed a second demographic trade-off along a ‘stature-fecundity’ axis that separated species with high recruitment rates and seedling performance from species with high growth and survival of larger individuals (≥ 1 cm dbh). The demographic space spanned by these trade-offs was almost perfectly aligned with the functional trait spectrum created from shade tolerance and a size dimensions (Rüger *et al.* in revision). This is consistent with the scaling of fundamental plant strategies from organs to individuals and populations. In order to test whether these results can be generalized to different forest we are requesting and have already (partially) assembled demographic data and functional traits from seeds to adult trees from nine CTFS plots (BCI, San Lorenzo, Fushan, Kenting, Luquillo, Yasuní, Pasoh, Palanan, Lambir).

Request

Pasoh 50-ha main census data (all trees dbh ≥ 1 cm), seedling and seed census data and tree species trait data (maximum height, wood density, seed mass, leaf area, leaf mass per area, leaf dry matter content, leaf phosphorous and nitrogen concentrations).

Project duration 1-2 years.

Planned visiting schedule No visit is planned.

Funding acknowledgement iDiv flexible pool project 34600559

Adler, P.B., HilleRisLambers, J. & Levine, J.M. (2007). A niche for neutrality. *Ecol. Lett.*, **10**: 95–104.

Bohlman, S. & Pacala, S. (2012). A forest structure model that determines crown layers and partitions growth and mortality rates for landscape-scale applications of tropical forests. *J. Ecol.*, **100:** 508–518.

Delchambre, L. (2014) Weighted principal component analysis: a weighted covariance eigendecomposition approach. *Mon. Not. R. Astron. Soc.* **446**: 3545–3555.

Rüger, N. *et al.* (in revision after invitation to re-submit) Beyond the fast-slow continuum: Demographic dimensions structuring a tropical tree community. *Ecol. Lett.*

Salguero-Gómez, R. *et al.* (2016) The fast-slow continuum and reproductive strategies structure plant life history variation worldwide. *PNAS,* **113**: 230–235.

Salguero-Gómez, R. (2017) Applications of the fast-slow continuum and reproductive strategy framework of plant life histories. *New Phytol.*, **213**, 1618–1624.

Stearns, S.C. (1999) The Evolution of Life Histories. Oxford Univ Press, New York, p 249.
